# Microbiome and Metabolome Analyses Reveal Novel Interplay Between the Skin Microbiota and Plasma Metabolites in Psoriasis

**DOI:** 10.3389/fmicb.2021.643449

**Published:** 2021-03-16

**Authors:** Dongmei Chen, Jingquan He, Jinping Li, Qian Zou, Jiawei Si, Yatao Guo, Jiayu Yu, Cheng Li, Fang Wang, Tianlong Chan, Huijuan Shi

**Affiliations:** ^1^Innovation Team for Skin Disease Diagnosis and Treatment Technology & Drug Discovery and Development, The General Hospital of Ningxia Medical University, Yinchuan, China; ^2^Institute of Human Stem Cell Research, The General Hospital of Ningxia Medical University, Yinchuan, China; ^3^Biotree Metabolomics Research Center, Biotree, Shanghai, China; ^4^Department of Oncology Surgery, Ningxia Medical University, Yinchuan, China; ^5^Clinical Medical School, Ningxia Medical University, Yinchuan, China; ^6^Department of Dermatovenereology, The General Hospital of Ningxia Medical University, Yinchuan, China

**Keywords:** psoriasis, skin microbiome, plasma metabolome, lipid metabolism, inflammation

## Abstract

Psoriasis is a chronic inflammatory skin disease that affects millions of people worldwide. There is still no effective approach for the clinical treatment of psoriasis. This is largely due to the lack of understanding of the pathological mechanism. Here, we comprehensively characterized the skin microbiome and plasma metabolome alterations of psoriasis patients. We observed that some pathogenic bacteria, including *Vibrio*, were significantly increased in psoriasis patients. The metabolomics results showed alterations in some metabolic pathways, especially pathways for lipid metabolism. In addition, microbiome-specific metabolites, including bile acids and kynurenine, were significantly changed. Correlation analysis revealed the interplay between the skin microbiota and plasma metabolites, especially between *Vibrio* and several lipids. Our results provide new evidence for the interplay between the skin microbiome and plasma metabolites, which is dramatically disrupted in psoriasis patients. This study also revealed the mechanism underlying the pathogenesis of psoriasis.

## Introduction

Psoriasis is one of the most common skin disorders worldwide, with approximately 2% of people affected (Boehncke and Schön, 2015). It affects not only the skin but also other organs. The molecular mechanism of psoriasis is not clear, which makes the discovery of new therapeutic drugs difficult. Most patients have to suffer from the disease for their whole life (Dubertret et al., 2006; Nestle et al., 2009).

The causes of psoriasis remain largely unknown but are reported to be related to many factors, including environmental factors, genetic factors, and immunologic factors (Nestle et al., 2009). In addition, the progression and even relapse after clinical treatment are all influenced by these factors, which act together and form the specific metabolic characteristics of psoriasis. Glucose metabolism, amino acid metabolism, and lipid metabolism have been shown to be significantly changed in psoriasis patients (Zeng et al., 2017; Zhu and Thompson, 2019). The roles of metabolic regulation of cell proliferation and apoptosis are considered to be key to unregulated keratinocyte pathogenesis in psoriasis (Luo et al., 2020; Pohla et al., 2020). In addition, it is well known that the chronic inflammatory features of psoriasis, the associated characteristics of metabolic syndrome with psoriasis, and even the diet-related pathogenesis mechanism of psoriasis all indicate the importance of metabolism in the disease (Wolters, 2005; Gisondi et al., 2018). These reports suggest that alterations in global metabolism may contribute to the specific phenotype of psoriasis patients. However, additional information is still needed to answer these questions.

Microorganisms, which are located in many sites in our body, play very important roles in system homeostasis. Most studies have focused on the gut microbiome. The abundance and composition of the gut microbiome can vary under different conditions and are related to many human diseases (Chávez-Talavera et al., 2017). Increasing evidence has suggested that the activity of the microbiota is critical, especially in modulating tissue metabolism (Liu et al., 2017; He et al., 2020). In recent years, the role of the microbiome in maintaining healthy skin status and regulating skin-related diseases has been reported (Zeeuwen et al., 2013; Dréno et al., 2016; Byrd et al., 2018). For psoriasis, the role of the gut microbiome in disease pathogenesis and progression has been reported. In addition, the gut microbiota can be a potential biomarker of the disease (Thio, 2018; Myers et al., 2019). However, the organization of the skin microbiome and its potential function in regulating global metabolism in psoriasis patients remain unclear.

In this study, we collected skin microbiome samples and plasma samples from patients with severe plaque psoriasis and from healthy controls. We performed 16S sequencing of the skin microbiome and plasma metabolomic analysis. Our results revealed alterations in the skin microbiota in psoriasis patients, including the accumulation of species of *Gammaproteobacteria*. Functional prediction revealed changes in metabolic pathways. In addition, our metabolomic data showed very obvious changes in systemic metabolism in psoriasis patients. Furthermore, we established a novel correlation map of the skin microbiome and plasma metabolites. These results highlighted the role of the skin microbiome in regulating global metabolism and provided new insights regarding the pathological view of psoriasis.

## Materials and Methods

### Patient Information

A total of 32 patients diagnosed with severe plaque psoriasis were recruited at the General Hospital of Ningxia Medical University (Ningxia Province, China) for this study from December 2018 to May 2019. At the same time, 29 healthy volunteers were recruited. The average age of the patients was 38.16 years, with a range of 17–74 years. For healthy controls, the average age was 35.53 years, with a range of 23–54 years. The severity of psoriasis was quantified by using the Psoriasis Area and Severity Index (PASI) score (38.96 ± 2.64, mean ± SE), the Psoriasis Global Assessment score (4.41 ± 0.13, mean ± SE), and the body surface area score (24.85 ± 2.82, mean ± SE). Patients who met the following criteria at the same time were included: patients with severe plaque psoriasis (PASI score ≥12) (Mrowietz et al., 2011), patients with at least half a year of disease duration, and patients who had previously received at least one course of systemic treatment without obvious improvement. The exclusion criteria were as follows: volunteers with severe liver or kidney damage, mental illness, hematopoietic dysfunction, or other serious organic disease; patients who received immunosuppressive treatment or high doses of glucocorticoids or retinoid treatment in the previous 2 months; and all participants, including healthy controls and psoriasis patients, who had used any skin care product or lotion in the previous week. None of the healthy volunteers had a history of any immune diseases, and none of them had any skin disorders. All samples and clinical information were obtained under the condition of informed consent. This study was conducted with the approval of the institutional review board of the General Hospital of Ningxia Medical University and in accordance with the Declaration of Helsinki.

### Sample Collection

Skin microbiome samples were collected according to a previous report (Oh et al., 2014). Briefly, a swab was rinsed with phosphate-buffered saline, and a defined skin area of approximately 2 × 2 cm^2^ was swabbed at least 20 times to maximize the amount of microbiome DNA collected. All samples were stored at −80°C until extraction.

The plasma samples were collected on the same day after overnight fasting with a heparin sodium anticoagulant tube. The samples were then centrifuged at 3,000 rpm for 10 min at room temperature. The supernatants were collected and aliquoted into different tubes and stored at −80°C.

### Microbiome DNA Extraction and 16S Sequencing

Genomic DNA from skin microbiome samples was extracted by using the Mobio Powersoil DNA Isolation Kit (Qiagen, Hilden, Germany) according to the manufacturer’s instructions. The V3 and V4 regions of the 16S rRNA genes were amplified by using Phusion^®^ High-Fidelity PCR Master Mix with GC Buffer (New England Biolabs, MA, United States) and the primers 341F and 806R. After purification of the polymerase chain reaction product by using AMPure XP magnetic beads (Beckman Coulter, IN, United States), the samples were analyzed by the Illumina NovaSeq 6000 platform (Illumina, CA, United States) through a paired-end sequencing strategy.

### 16S Sequencing Data Analysis

After Illumina sequencing, barcode and primer sequences were removed. Specific tags were generated by FLASH software^1^ according to the overlap information of the reads. Then, we applied Trimmomatic software (v0.33) to remove the low-quality tags and obtained clean tags. Clean tags were further filtered to exclude the chimeric sequences by using UCHIME software (v4.2). Next, the remaining sequences with an identity >97% were classified as operational taxonomic units (OTUs) by using Uparse software^2^. Taxonomic information was annotated by searching against the SSU rRNA database^3^. OTUs were then assigned to different phylogenetic levels (kingdom, phylum, class, order, family, genus, and species). Alpha diversity and beta diversity were analyzed by QIIME software (v1.9.1) based on the effective tags. The relative abundance and the difference in diversity were compared by Student *t*-test and the Wilcoxon rank-sum test. Furthermore, linear discriminant analysis coupled with effect size (LEfSe) was applied to identify microorganisms that can be used to discriminate psoriasis patients from people with no psoriasis.

### Liquid Chromatography–Mass Spectrometry Metabolomic Data Collection

A 100-μL plasma sample from each patient was mixed with 300 μL of methanol containing 1 μg/mL 2-chloro-L-phenylalanine (Hengbai Biotech, Shanghai, China) as the internal standard. After brief sonication in ice water for 10 min, all the samples were placed at −40°C for 1 h and centrifuged at 10,000 rpm for 15 min at 4°C. Then, the samples were resuspended in 100 μL of 50% acetonitrile. For quality control (QC) sample preparation, a mixture containing an equal volume (10 μL) of each plasma extract was prepared.

For liquid chromatography–mass spectrometry (LC-MS) metabolomic data collection, all plasma samples were analyzed by a 1290 UHPLC instrument (Agilent Technologies, CA, United States) coupled with a Thermo Q Exactive Focus (Thermo Fisher Scientific, MA, United States) by Biotree Ltd. (Shanghai, China), according to previously reported methods with minor modifications (He et al., 2020). Briefly, mobile phase A in positive ion mode was 0.1% formic acid in water, and in negative ion mode, it was 5 mmol/L ammonium acetate in water. Mobile phase B was acetonitrile. The elution gradient was set as follows: 1% B at 1 min, 99% B at 8 min, 99% B at 10 min, 1% B at 10.1 min, and 1% B at 12 min. The flow rate was set to 0.5 mL/min. The Q Exactive mass spectrometer was run at a spray voltage of 4.0 kV in positive mode and −3.6 kV in negative mode. Other ESI source conditions were as follows: sheath gas flow rate of 45 Arb, Aux gas flow rate of 15 Arb, and capillary temperature of 400°C. All MS1 and MS2 data were obtained under the control of Xcalibur (Thermo Fisher Scientific). A UPLC HSS T3 column (Waters, MA, United States) was used for all analyses. Organic reagents, including methanol, acetonitrile, and formic acid (HPLC grade), were purchased from CNW Technologies (Dusseldorf, Germany).

### Gas Chromatography–MS Data Collection

The extracted plasma samples were resuspended in 30 μL of methoxyamine hydrochloride (20 mg/mL in pyridine) and incubated at 80°C for 30 min. After derivatization with 40 mL of N,O-bis(trimethylsilyl)trifluoroacetamide (BSTFA) with 1% trimethylsilyldiethylamine (Sigma, Darmstadt, Germany) at 70°C for 1.5 h, the samples were cooled down gradually to room temperature. For QC sample preparation, a mixture containing an equal volume (10 μL) of each plasma extract was prepared. An additional 5 μL of saturated fatty acid methyl esters (Dr. Ehrenstorfer GmbH, Augsburg, Germany) dissolved in chloroform was added to the QC samples for gas chromatography (GC)–MS analysis.

Gas chromatography–time of flight (TOF)–MS analysis was carried out by using an Agilent 7890 gas chromatograph (Agilent Technologies) coupled with a Pegasus HT TOF mass spectrometer (LECO, Michigan, United States). In this analysis, a DB-5MS capillary column (30 m × 250 μm × 0.25 μm, Agilent Technologies) was used. The carrier gas was helium, the front inlet purge flow was set as 3 mL/min, and the gas flow rate was 1 mL/min. The temperature gradient was set as 50°C for 1 min, increased to 310°C at a rate of 20°C/min, and then maintained for 6 min. The front injection temperature, transfer line temperature, and ion source temperature were 280, 280, and 250°C, respectively. The energy was −70 eV in electron impact mode. The MS data were acquired in full-scan mode with an m/z range of 50–500 at a rate of 12.5 spectra per second after a solvent delay of 4.85 min. A 1-μL sample was injected for this analysis.

### LC-MS and GC-MS Metabolomic Data Analyses

ProteoWizard software was used to transform the original LC-MS data to mzXML format. The data were processed by XCMS. GC-MS raw data were processed by Chroma TOF software. After peak identification, peak alignment, peak extraction, retention time (RT) correction, and peak integration, a three-dimensional data matrix was obtained. To make the metabolomics data reproducible and reliable, peaks with relative standard deviations greater than 30% in the QC samples were filtered out. The remaining peaks were identified by comparison of RT and mass to charge ratio (m/z) indexes in a library containing spectral information from the online database of HMDB^4^, Kyoto Encyclopedia of Genes and Genomes (KEGG)^5^, and the in-house library. The GC-MS data were matched with the LECO-Fiehn Rtx5 database. Peak intensity was quantified by using the area under the curve. The data matrix was further processed by removing the peaks with missing values in more than 50% of the samples and substituting the remaining missing values with half of the minimum value. Then, a new data matrix was generated by normalizing the data to the peak intensity of the internal standard.

### Statistical Analysis

Statistical analysis was performed by using Microsoft Excel (Microsoft Inc., Redmond, WA, United States) and R software version 3.5.1 (R Foundation for Statistical Computing, Vienna, Austria). The differential abundance of bacterial taxa at different levels (phylum, class, order, family, and genus) between psoriasis patients and healthy controls was calculated by the Wilcoxon rank-sum test and Metastat. The differences in alpha diversity indexes were determined by Student *t*-test. The beta diversity difference between psoriasis patients and the control group was analyzed by analysis of similarity (ANOSIM). To understand the difference in the metabolomic profile between psoriasis patients and healthy people, multivariate statistical analyses, including principal component analysis (PCA) and orthogonal projections to latent structure-discriminant analysis (OPLS-DA), were carried out. Small molecules with a VIP (variable importance in projection) >1 in OPLS-DA and *p* < 0.05 by Student *t*-test were considered significantly altered metabolites. Spearman correlation was carried out to determine the relationship between the skin microbiota and plasma metabolites.

## Results

### Altered Skin Microbiota Composition in Psoriasis Patients

We recruited 32 severe plaque psoriasis patients (PASI > 12) and 29 healthy controls to identify the psoriasis-related microbiota. After QC, the DNA sample amounts from only 26 patients and 10 controls were sufficient for 16S sequencing.

**FIGURE 1:**
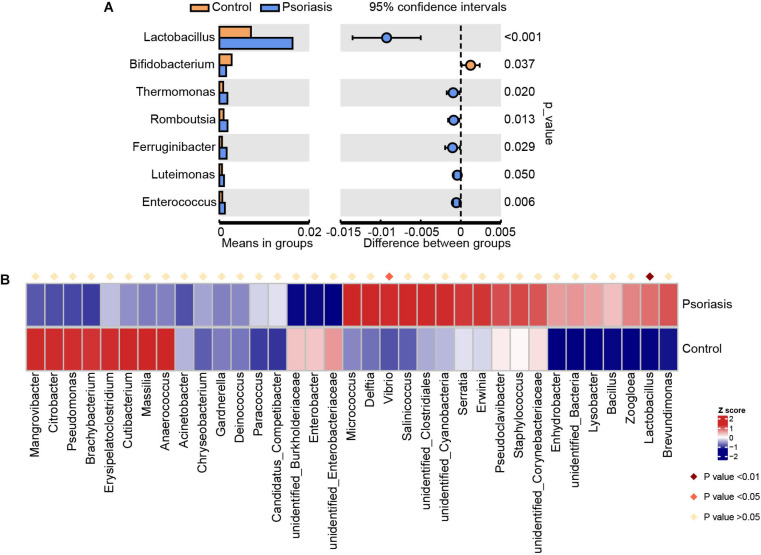
Skin microbiota alterations in psoriasis patients. **(A)** Significantly altered skin microbiota in psoriasis patients compared with healthy controls analyzed by Student’s *t*-test at the genus level. **(B)** Heatmap shows the significantly altered skin microbiota at the genus level in psoriasis patients compared with healthy controls analyzed by Metastat.

Overall, we obtained 83,998 effective tags and 7,887 OTUs according to 97% similarity. After taxonomic assignment against the Silva132 database, 7,606 OTUs were annotated at different phylogenetic levels (Supplementary Table 1). According to the species accumulation curve, the sequencing data and samples were sufficient for taxon identification. However, there were no significant differences between control individuals and psoriasis patients in terms of number of species on skin (Supplementary Figure 1A). In addition, the alpha diversity indexes, including the total observed species, Shannon index, ACE index, Simpson index, and Chao1 index, of the skin of psoriasis patients were not significantly different from those of the control group (Supplementary Figures 1B–F). To identify the microbes that were altered in psoriasis patients, we then conducted Student *t* test at the genus level (Figure 1A). The average abundance of *Lactobacillus*, which is widely distributed in the human gut and skin and plays a role as a lactic acid producer, was increased in psoriasis patients. This may suggest a potential positive role of *Lactobacillus* in regulating skin cell proliferation, which is consistent with a previous report that *Lactobacillus* was capable of enhancing skin repair after UV damage (Im et al., 2018). Moreover, the abundances of *Thermomonas* and *Luteimonas*, which are pathogenic members of Proteobacteria (phylum)_Gammaproteobacteria (class), were also increased, suggesting that the skin of psoriasis patients was a pathogenic environment. To further analyze the alterations in the microbiota in psoriasis patients, we applied Metastat, another widely used statistical analysis tool, to screen for significantly changed organisms (Figure 1B). Similarly, the change in the *Lactobacillus* abundance was also identified as an important alteration. In addition, another member of *Gammaproteobacteria*, *Vibrio*, was identified as being significantly elevated in psoriasis patients. Together, these data suggest an elevation in the abundance of pathogenic bacteria, especially *Gammaproteobacteria*, in psoriasis patients.

**FIGURE 2:**
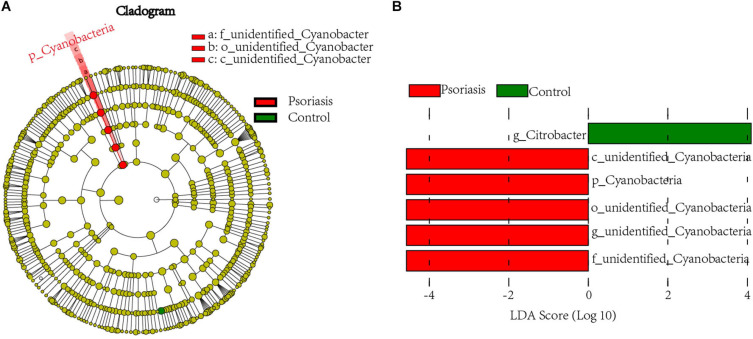
Linear discriminant analysis (LDA) effect size. **(A)** Cladogram of LEfSe of the skin microbiome from 16S sequencing results. Red and green circles represent the differences of the most abundant microbiome class. The diameter of each circle is proportional to the relative abundance of the taxon. **(B)** Histogram of the LDA scores for differentially abundant microbes in psoriasis patients and healthy controls. Red, enriched in psoriasis patients; green, enriched in healthy controls.

To further examine the alterations associated with psoriasis, we conducted LEfSe analysis. The main differences were the increase in abundance of undefined_Cyanobacteria (class unidentified_Cyanobacteria and order Cyanobacteria) in psoriasis patients (Figure 2A). Some differences were also observed at a lower taxonomic level. Psoriasis patients showed a loss in the abundance of the genus *Citrobacter* (Figure 2B). Taken together, these data indicate alterations in the commensal gut microbiome composition in psoriasis patients, suggesting dysregulation of the microbial community.

### Functional Prediction of the Skin Microbiome of Psoriasis Patients

**FIGURE 3:**
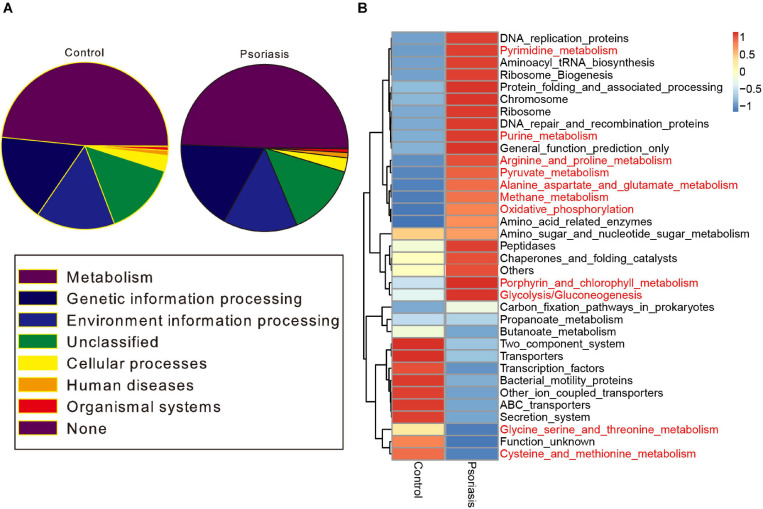
Functional prediction of the skin microbiome. **(A)** KEGG pathway classification of the annotated taxa in healthy controls and psoriasis patients. **(B)** Heatmap showing the altered metabolic pathways in psoriasis patients compared with healthy controls. Red represents upregulation, and blue represents downregulation.

To further determine the functional impact of skin gut microbiota alterations in psoriasis, we predicted the KEGG pathways based on the 16S sequencing data by using PICRUSt software (Langille et al., 2013). Metabolic pathways ranked as the most abundant pathways predicted, accounting for approximately 50% of the pathways (Figure 3A). Among these pathways, carbohydrate metabolism and the metabolism of other amino acids were obviously decreased (Supplementary Figure 2). In contrast, pyrimidine and purine metabolism (nucleotide metabolism); glycolysis/gluconeogenesis, oxidative phosphorylation, and methane metabolism (energy metabolism); the metabolism of cofactors and vitamins; and the biosynthesis of other secondary metabolites showed an increase in psoriasis patients compared with healthy controls (Figure 3B and Supplementary Figure 2).

### Plasma Metabolic Profiling of Psoriasis Patients

**FIGURE 4:**
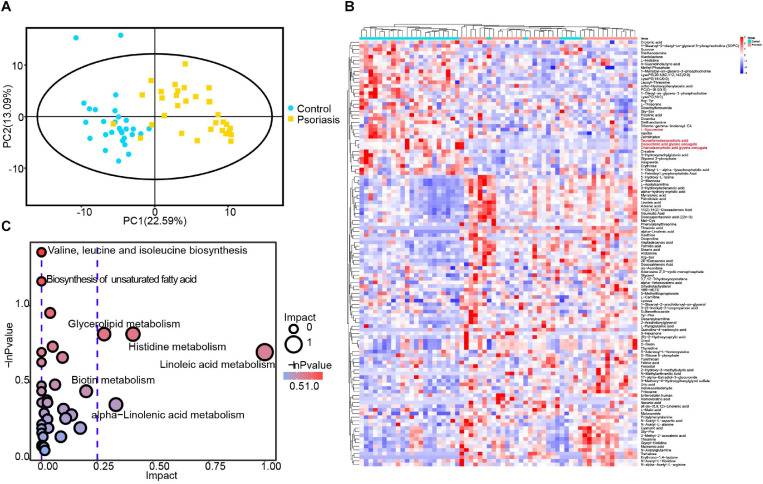
Plasma metabolomic profiling. **(A)** PCA revealed clear separation of the plasma metabolome between psoriasis patients and healthy controls. **(B)** Heatmap showing the significantly changed metabolites in psoriasis patients compared with healthy controls. The metabolites labeled with red font are microbiota-specific metabolites. **(C)** Bubble plot of the metabolic pathway enrichment analysis results.

Microbiota alterations have been reported to be correlated with tissue metabolism in many studies (Liu et al., 2017; Olson et al., 2018). In addition, our data showed that skin microbiota–mediated small molecule metabolism was impacted by psoriasis (Figure 3). We thus investigated the metabolic alterations in psoriasis patients by applying GC and ultrahigh-pressure LC coupled with MS. In general, a total of 3,562 features and 716 metabolites were obtained (Supplementary Table 2). PCA showed very obvious separation of metabolic profiles between psoriasis patients and healthy controls, demonstrating different metabolic activities (Figure 4A). After statistical analysis, we obtained 117 significantly altered metabolites (VIP >1 and *p* < 0.05) (Figure 4B). Among them, we found several microbiome-generated metabolites that were also significantly changed. These included taurochenodeoxycholic acid (TCDCA), deoxycholic acid glycine conjugate (GDCA), chenodeoxycholic acid glycine conjugate, and L-kynurenine (Chávez-Talavera et al., 2017; Agus et al., 2018; He et al., 2020). To uncover the metabolic pathway alterations, we conducted KEGG pathway analysis of the differentially expressed metabolites by using Metaboanalyst^6^ (Figure 4C). Branched-chain amino acid metabolism (valine, leucine, and isoleucine biosynthesis), which was reported to be closely related to microbiota metabolic activity (Liu et al., 2017), was significantly altered. In addition, the metabolism of α-linolenic acid and linoleic acid, which reflect the inflammation status of tissues (Sergeant et al., 2016), was also significantly altered.

### Novel Interplay Between the Skin Microbiota and Plasma Metabolism

**FIGURE 5:**
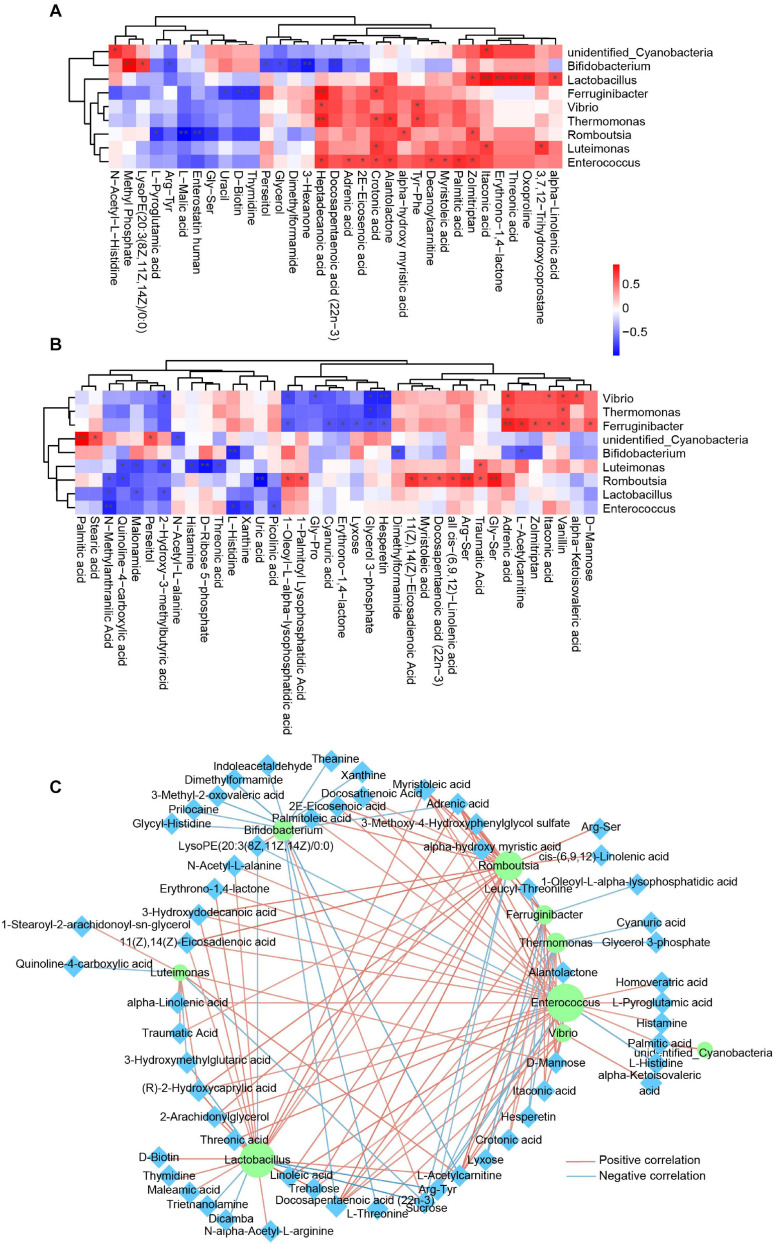
Integrated analysis of skin microbes and plasma metabolites. **(A)** Spearman correlation analysis between significantly altered skin microbes and significantly changed plasma metabolites in the healthy control group. Red, positive correlation; blue, negative correlation. *p < 0.05. **(B)** Spearman correlation analysis between significantly altered skin microbes and significantly changed plasma metabolites in the psoriasis group. Red, positive correlation; blue, negative correlation. *p < 0.05. **(C)** Spearman correlation network between significantly altered skin microbes and significantly changed plasma metabolites in all samples from both groups. The purple circle represents the skin microbiota, and the cyan diamond represents plasma metabolites. Red line, positive correlation; blue line, negative correlation. Only the correlations with *p* < 0.05 are shown. **p < 0.01.

Many articles have reported the correlation of the gut microbiota and blood metabolism (Liu et al., 2017; He et al., 2020), whereas little is known about the relationship of the skin microbiota and blood metabolism. In this study, we carried out Spearman correlation analysis of the annotated skin microbiota at the genus level and the identified plasma metabolites. The association of the skin microbiota and plasma metabolites was different between healthy controls and psoriasis patients (Figures 5A,B), suggesting that the alteration of plasma metabolites was closely related to the skin microbiome. Interestingly, most of the associations between *Lactobacillus* and plasma metabolites and the associations between *Enterococcus* and plasma metabolites in healthy controls (Figure 5A) disappeared in psoriasis patients (Figure 5B). In addition, new correlations between *Vibrio*, *Ferruginibacter*, *Romboutsia*, and plasma metabolites were established in psoriasis patients (Figure 5B). The metabolites that showed a significant positive association with specific skin bacteria in both healthy controls and psoriasis patients were itaconic acid, crotonic acid, and heptadecanoic acid, which are involved in lipid metabolism (Figures 5A,B). Notably, some plasma metabolites were negatively associated with the skin microbiota in psoriasis patients. In addition to several lipids, xanthine, D-ribose 5-phosphate, and uric acid participate in nucleotide metabolism (Figure 5B). These results suggest a role of the skin microbiota in influencing lipid and nucleotide metabolism in psoriasis patients.

To determine a global relationship between the skin microbiota and plasma metabolism, we then conducted Spearman correlation analysis by using all the samples from healthy controls and systemic lupus erythematosus (SLE) patients. The correlations were shown in a Cytoscape network (Figure 5C), further suggesting a fundamental relationship of the skin microbiota and plasma metabolism. Interestingly, receiver operating characteristic (ROC) curve analysis revealed that many skin microbiota–associated plasma metabolites are potential biomarkers for SLE classification (Supplementary Figure 3).

## Discussion

Previous studies investigated the changes in the blood metabolome and gut microbiome that occur in psoriasis. However, it is not sufficient to understand the pathogenesis of psoriasis, as the disease primarily occurs on the skin. Here, we analyzed skin microbiota alterations in psoriasis patients by using 16S sequencing and plasma metabolomic changes by applying an LC-MS metabolomics approach. According to our results, the plasma metabolic homeostasis of psoriasis patients was disrupted and was correlated with alterations in the skin microbiome. We further identified some skin microbes at the genus level, such as *Enterococcus* and *Vibrio*, which are critical for plasma metabolism in psoriasis patients. In addition, we also identified some skin microbiota–associated plasma metabolites that are potential biomarkers for strongly discriminating healthy controls from psoriasis patients.

In our untargeted metabolomic study, many plasma metabolites were significantly changed in psoriasis patients. Pathway analysis revealed enrichment in both amino acid metabolism and lipid metabolism pathways (Figure 4). The valine, leucine, and isoleucine biosynthesis pathway, which is a branched-chain amino acid metabolism pathway mediated by the microbiota, has been reported to be related to many diseases (Liu et al., 2017, 2020). In addition, several lipid metabolism pathways were also enriched, including biosynthesis of unsaturated fatty acids, glycerolipid metabolism, linoleic acid metabolism, and α-linolenic acid metabolism. Glycerolipids that play a very important role in membrane mobility and provide building blocks for membrane biogenesis have also been reported previously as potential diagnostic biomarkers in psoriasis patients (Zeng et al., 2017). Furthermore, the alteration in linoleic acid and α-linolenic acid metabolism reflects the inflammation status of psoriasis (Boehncke, 2018). In addition, the microbiota-mediated metabolism of bile acids (TCDA, TCDCA, and GDCA) is well known for its role in lipid metabolism, and L-kynurenine is well known for its role in inflammation regulation (Vítek and Haluzík, 2016; Cervenka et al., 2017). Altogether, the metabolomic results indicate the role of the microbiota in the regulation of lipid metabolism and the inflammatory response in psoriasis patients.

Psoriasis is a chronic inflammatory skin disorder. Increasing evidence has suggested the role of the skin microbiome in the pathogenesis of diseases (Grice, 2014; Picardo and Ottaviani, 2014; Li et al., 2019). In our study, some skin microbes were significantly altered in psoriasis patients compared with healthy people, which is consistent with previous research (Chang et al., 2018). Among them, the bacteria *Thermomonas* and *Luteimonas* from the pathogenic Proteobacteria (phylum) and Gammaproteobacteria (class) were significantly increased, suggesting the pathological role of these bacteria in psoriasis. *Vibrio*, the most strongly and significantly increased bacterial taxon (Figure 1B), is well known for its role as the cholera pathogen (Conner et al., 2016). Because the species of *Vibrio* mainly live in seawater or brackish water (Vasagar et al., 2018), people should be very careful when consuming seafoods or when exposed to seawater. These results indicate that the accumulation of the pathogenic microbiota is a possible reason for the pathogenesis of psoriasis. This conclusion was also confirmed by functional analysis of the skin microbiota. Skin microbiome–mediated nucleotide metabolism and amino acid metabolism activities were elevated in psoriasis patients compared with healthy controls (Figure 3B), as small-molecule metabolism pathways are critical for providing building blocks for skin cell proliferation.

The interaction between skin microorganisms and blood metabolism has rarely been investigated. In this article, we analyzed the Spearman correlation of significantly altered skin microbes and significantly changed plasma metabolites (Figure 5). The results highlighted the role of the skin microbiota in the regulation of plasma metabolism, especially the role of the pathogens *Enterococcus* and *Vibrio*. These data also suggest the role of the skin microbiome in skin homeostasis, which is critical for the maintenance of the immunological barrier of skin (Belkaid and Tamoutounour, 2016).

In summary, our study integrating skin microbiome 16S sequencing and plasma metabolomic data reveals alterations in global metabolic homeostasis status and the association of the skin microbiota with psoriasis. Considering the impact of many factors, including race, ethnicity, lifestyle, and environmental factors, on the skin microbiome and global metabolome, more studies are needed to address the role of the skin microbiome in the pathogenesis of psoriasis. In addition, additional studies are needed to understand the key skin microbes involved in the pathogenesis of psoriasis, especially through an effect on global metabolism. Our data provide the underlying mechanism of skin microbiome–mediated regulation of blood metabolism in patients with psoriasis. The results will be helpful for understanding the pathological mechanism of psoriasis.

## Data Availability Statement

The datasets presented in this study can be found in online repositories. The names of the repository/repositories and accession number(s) can be found below: EBI metagenomics, accession no: PRJEB42803 (ERP126714).

## Ethics Statement

The studies involving human participants were reviewed and approved by the Institutional Review Board of the General Hospital of Ningxia Medical University. Written informed consent to participate in this study was provided by the participants’ legal guardian/next of kin.

## Author Contributions

HS designed and supervised the study and edited the manuscript. HS, JL, QZ, JS, YG, JY, CL, and FW collected the clinical samples and determined the clinical measurement indexes. HS and QZ analyzed the clinical indicators. DC, JH, TC, and HS performed the metabolomic, 16S sequencing and bioinformatic analyses. HS, JH, and DC interpreted the data and wrote the manuscript. All authors contributed to the article and approved the submitted version.

## Conflict of Interest

JH and TC are employed by the company Shanghai Biotree Biomedical Biotechnology co., LTD. The remaining authors declare that this study was conducted in the absence of any commercial or financial relationships.

## Footnotes

1 http://ccb.jhu.edu/software/FLASH/, v1.2.7

2 http://www.drive5.com/uparse/, v7.0.1001

3 www.Arb-silva.de

4 www.hmdb.ca

5 www.genome.jp/kegg

6 www.metaboanalyst.ca

## Supplementary Material

Supplementary Figure 1 Alpha diversity indexes of the skin microbiome in psoriasis patients and healthy controls. **(A)** Curve of accumulated species number in psoriasis patients and healthy controls. **(B–F)** Alpha diversity indexes, including observed species **(B)**, ACE index **(C)**, Shannon index **(D)**, Simpson index **(E)**, and Chao1 index **(F)**, in psoriasis patients and healthy controls.

Supplementary Figure 2 Predicted function of the skin microbiome in level 2. The function of the skin microbiome from healthy controls and psoriasis patients was predicted and plotted. Red represents increased pathway annotation. Blue indicates reduced pathway annotation. Metabolic pathways are labeled with red font.

Supplementary Figure 3 ROC curve of the metabolites significantly associated with the skin microbiota. Biomarker analysis of the metabolites significantly correlated with the skin microbiota in Figure 5 shows the high AUCs. Only the metabolites with AUC value >0.8 are shown.

Supplementary Table 1 The relative abundance of the annotated skin microbiota measured by 16S sequencing.

Supplementary Table 2 The relative abundance of the identified plasma metabolites measured by the LC-MS metabolomic approach.
