# Supplementary figures and images for: Microbiome and Metabolome Analyses Reveal Novel Interplay Between the Skin Microbiota and Plasma Metabolites in Psoriasis
Source: Front Microbiol. 2021 Mar 16;12:643449. doi: 10.3389/fmicb.2021.643449 (PMC8007969; doi:10.3389/fmicb.2021.643449)

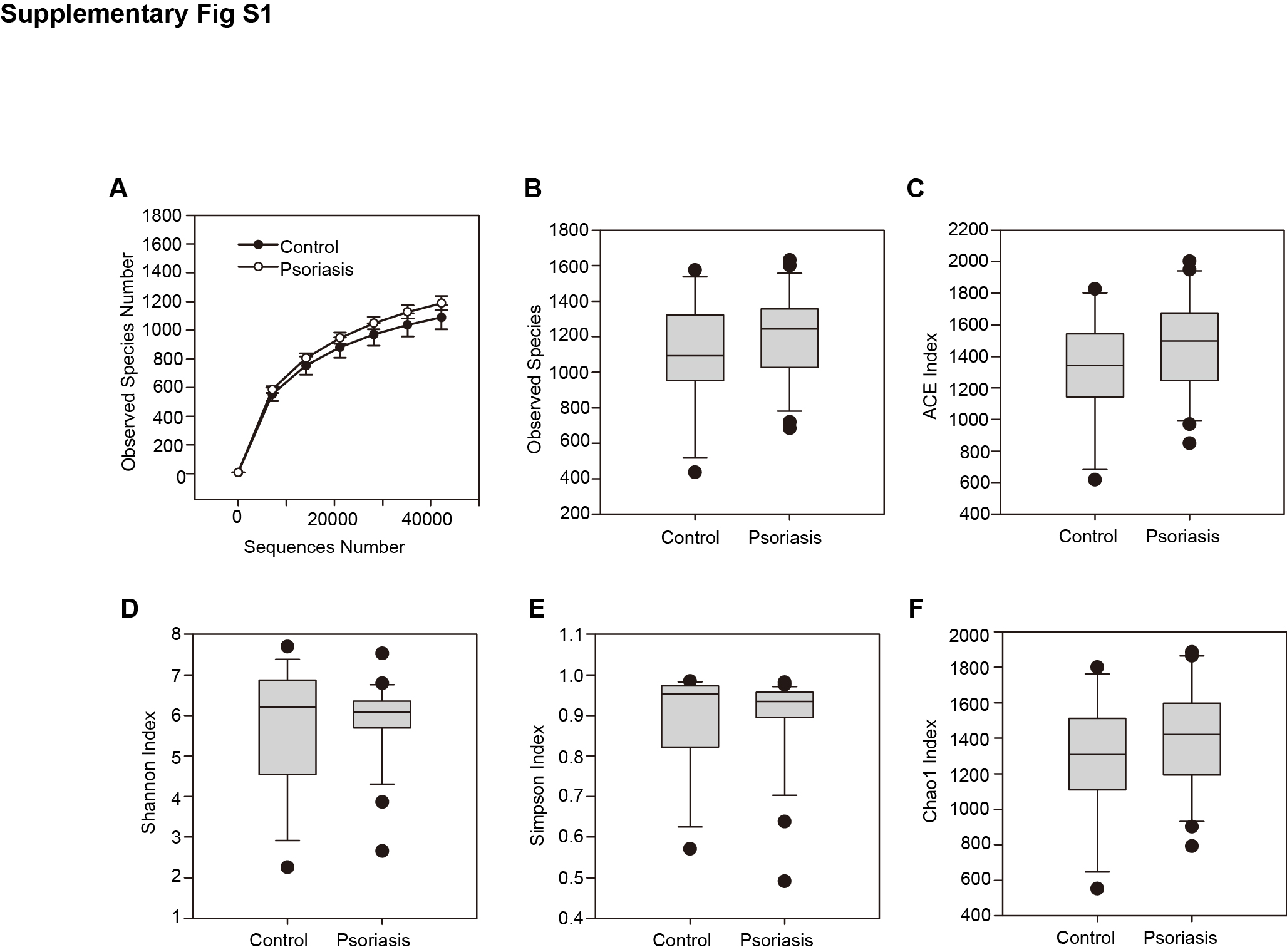

Supplement: Supplementary Figure 1 — Alpha diversity indexes of the skin microbiome in psoriasis patients and healthy controls. (A) Curve of accumulated species number in psoriasis patients and healthy controls. (B–F) Alpha diversity indexes, including observed species (B), ACE index (C), Shannon index (D), Simpson index (E), and Chao1 index (F), in psoriasis patients and healthy controls. [file Image_1.JPEG]

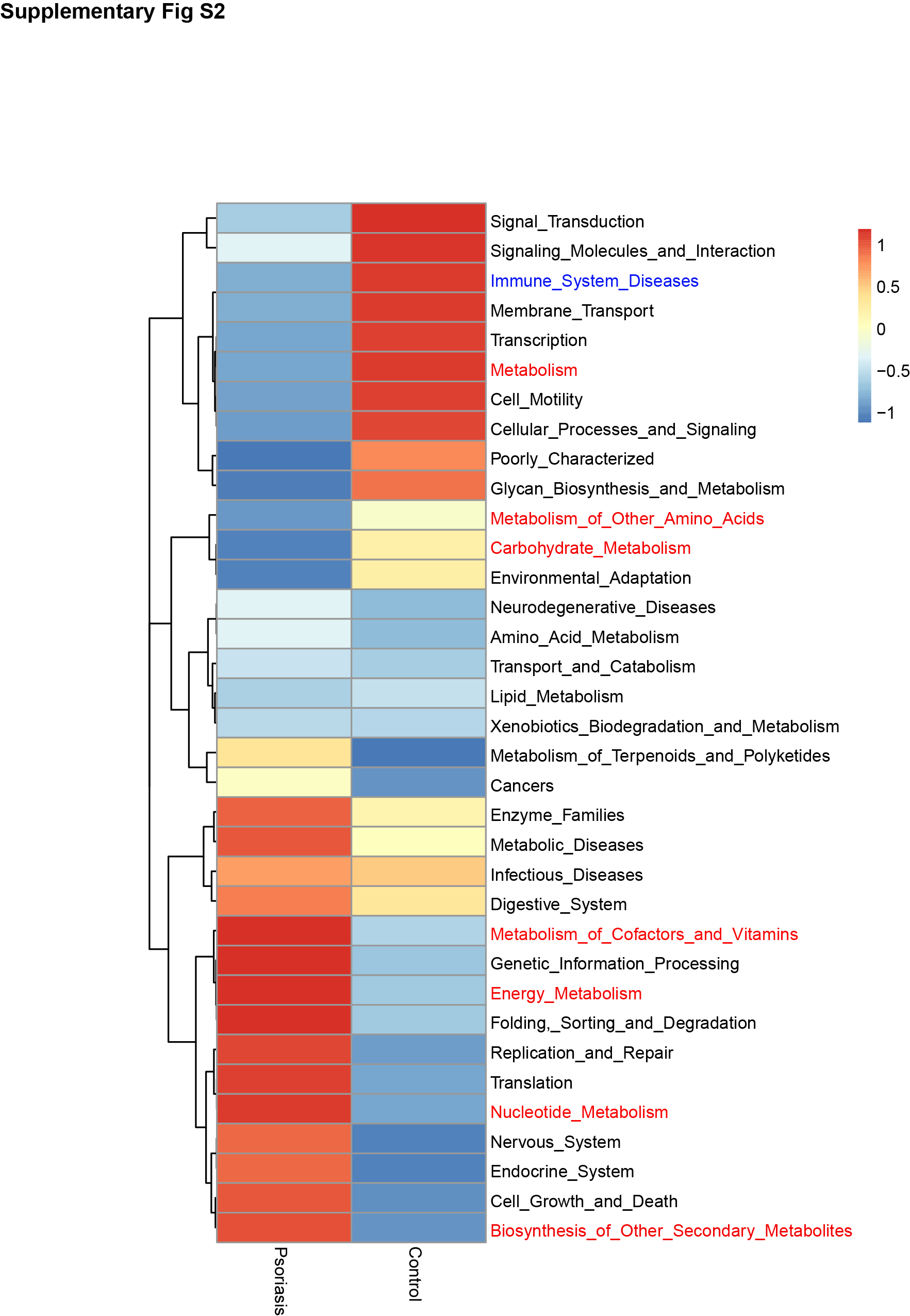

Supplement: Supplementary Figure 2 — Predicted function of the skin microbiome in level 2. The function of the skin microbiome from healthy controls and psoriasis patients was predicted and plotted. Red represents increased pathway annotation. Blue indicates reduced pathway annotation. Metabolic pathways are labeled with red font. [file Image_2.JPEG]

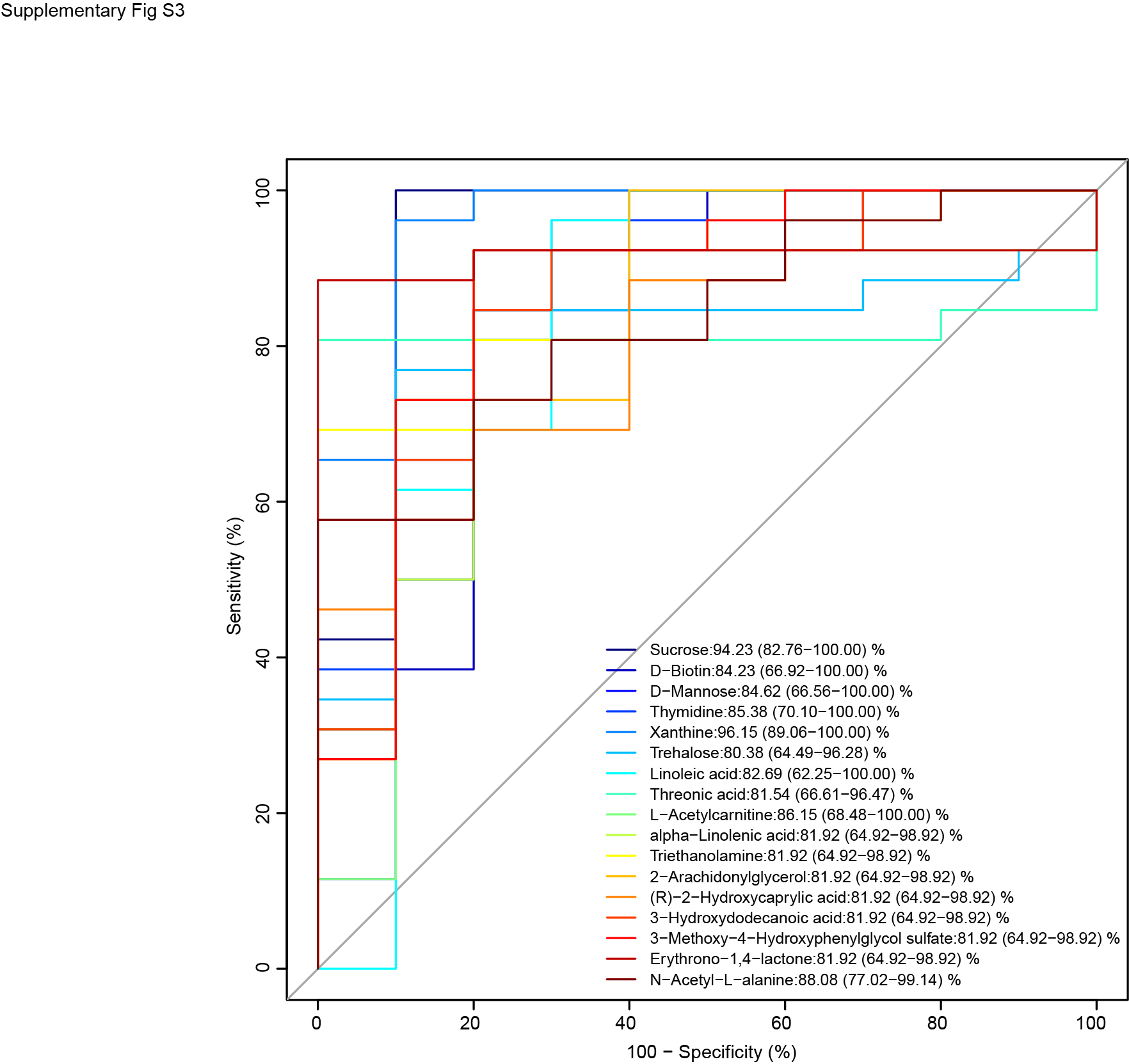

Supplement: Supplementary Figure 3 — ROC curve of the metabolites significantly associated with the skin microbiota. Biomarker analysis of the metabolites significantly correlated with the skin microbiota in Figure 5 shows the high AUCs. Only the metabolites with AUC value >0.8 are shown. [file Image_3.JPEG]
